# Age-related mushroom body expansion in male sweat bees and bumble bees

**DOI:** 10.1038/s41598-021-96268-w

**Published:** 2021-08-23

**Authors:** Mallory A. Hagadorn, Karlee Eck, Matthew Del Grosso, Xavier Haemmerle, William T. Wcislo, Karen M. Kapheim

**Affiliations:** 1 Department of Biology, Utah State University, 5305 Old Main Hill, Logan, UT 84322 USA; 2 Smithsonian Tropical Research Institute, 0843-03092 Panama City, Republic of Panama

**Keywords:** Developmental biology, Neuroscience, Social evolution

## Abstract

A well-documented phenomenon among social insects is that brain changes occur prior to or at the onset of certain experiences, potentially serving to prime the brain for specific tasks. This insight comes almost exclusively from studies considering developmental maturation in females. As a result, it is unclear whether age-related brain plasticity is consistent across sexes, and to what extent developmental patterns differ. Using confocal microscopy and volumetric analyses, we investigated age-related brain changes coinciding with sexual maturation in the males of the facultatively eusocial sweat bee, *Megalopta genalis,* and the obligately eusocial bumble bee, *Bombus impatiens*. We compared volumetric measurements between newly eclosed and reproductively mature males kept isolated in the lab. We found expansion of the mushroom bodies—brain regions associated with learning and memory—with maturation, which were consistent across both species. This age-related plasticity may, therefore, play a functionally-relevant role in preparing male bees for mating, and suggests that developmentally-driven neural restructuring can occur in males, even in species where it is absent in females.

## Introduction

Some structural and functional brain changes (i.e., neuroplasticity) occur independent of experience, as a natural part of development^1^. This age-related, ‘experience-expectant’ neuroplasticity likely primes neural systems to anticipate predictable life events^2,3^. Age-related expansion of key brain regions have been well documented in many highly social Hymenoptera, including ants^4,5^, bees^6–13^, and wasps^14,15^. These studies, however, have focused almost exclusively on females^16,17^, and it is unclear to what extent age-related neuroplasticity occurs in males. The function and drivers of age-related brain plasticity are likely different for males and females, and a female-biased focus could thus limit our understanding of how and why neuroplasticity evolves.

We redressed this bias by characterizing age-related neuroplasticity in the males of two bee species within the timespan of reproductive maturation. Cognitive demands prior to nest departure may be minimal in males, which could be accompanied by delayed neural development early in life. However, activities associated with mating and reproductive success likely require enhanced cognitive abilities^17^. For example, learning odors to avoid in-breeding with close kin, identifying individual females, remembering previously non-receptive mates, orienting spatially and navigating between aerial leks, as well as specializing on certain reproductive tactics may all require adaptive neural reorganization with male maturation^18–24^.

We investigated the effects of age on neuroplasticity in males of two bee species. In the facultatively eusocial sweat bee *Megalopta genalis* (Halictidae), females nest either solitarily or in small groups^25^. These social strategies tend to differ in patterns of sex ratio investment^26^, but all females are potential mates for males. In the obligately eusocial bumble bee *Bombus impatiens* (Apidae), males mate only with reproductive gynes and not the abundant workers present among large colonies^27^. In both species, males remain in the nest for at least a few days following eclosion, and eventually leave or are ejected, presumably as they become reproductively mature^27,28^. This period may also be accompanied by neural maturation, but this has never been investigated. To test this hypothesis, we compared mushroom body volumes of aged, mature males relative to young, newly eclosed males. Mushroom bodies are neuropil in the insect brain associated with sensory integration, learning, and memory^29^. If neuroplasticity coincides with reproductive maturation, then mushroom bodies should expand with age, independent of experience.

## Results

**Figure 1:**
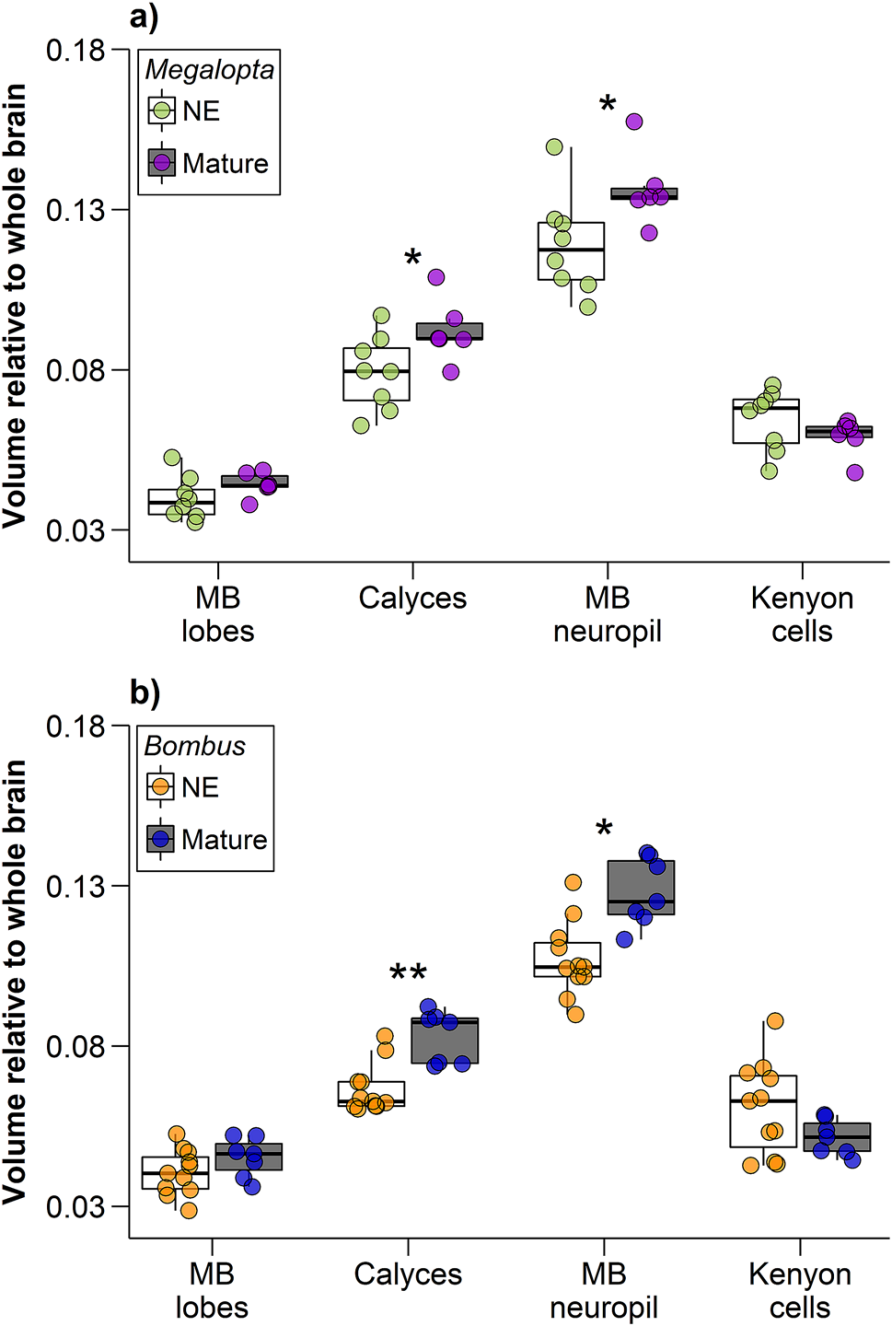
Mushroom body (MB) expansion occurs with maturation in male bees. Relative volumes of the mushroom body structures as whole brain proportions for (**a**) *M. genalis* and (**b**) *B. impatiens.* In both species, mature males had larger calyces (lip + collar + basal ring; *M. genalis*: t =  − 2.23, df = 12, p = 0.046; *B. impatiens*: t =  − 4.34, df = 16, p = 0.0005) and mushroom body neuropil (peduncles + lobes + calyces; *M. genalis*: t =  − 2.30, df = 12, p = 0.04; *B. impatiens*: t =  − 3.84, df = 16, p = 0.001) relative to newly eclosed bees. Dots represent individual data points for newly eclosed (NE; white boxes; *M. genalis* green dots, *N* = 8; *B. impatiens* orange dots, *N* = 11) and mature (gray boxes; *M. genalis* purple dots, *N* = 6; *B. impatiens* blue dots, *N* = 7) males. Boxes indicate interquartile range, lines are medians, and whiskers extend to 1.5 the interquartile range. “*” = unadjusted *p* < 0.05 and “**” = unadjusted *p* < 0.001.

**Figure 2:**
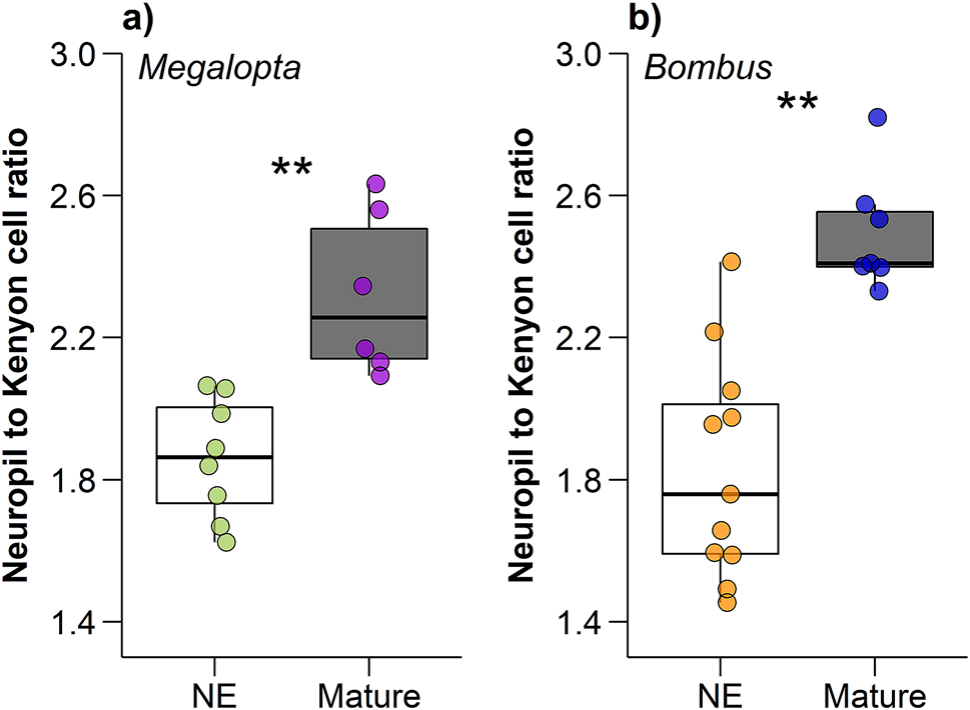
Age-related neuroplasticity in male bees. Neuropil:Kenyon cell ratios are significantly higher in mature, relative to newly eclosed, (**a**) *M. genalis* (t =  − 4.32, df = 12, p = 0.001) and (**b**) *B. impatiens* (t =  − 5.12, df = 16, p = 0.0001) bees. Dots represent individual data points for newly eclosed (NE; white boxes; *M. genalis* green dots, *N* = 8; *B. impatiens* orange dots, *N* = 11) and mature (gray boxes; *M. genalis* purple dots, *N* = 6; *B. impatiens* blue dots, *N* = 7) males. Boxes indicate interquartile range, lines are medians, and whiskers extend to 1.5 the interquartile range. “*” = unadjusted *p* < 0.05 and “**” = unadjusted *p* < 0.001.

Males of both *M. genalis* and *B. impatiens* exhibit age-related expansion of the mushroom bodies. In *M. genalis*, the relative volumes of calyces were 16.6% higher in mature males than in newly eclosed males (Fig. 1a; t =  − 2.23, df = 12, p = 0.046, Hedges’ *g* = 1.20). Total mushroom body neuropil was 14.7% larger in mature males than in newly eclosed males (Fig. 1a; t =  − 2.30, df = 12, p = 0.040, Hedges’ *g* = 1.24). Although, after adjusting for multiple comparisons, these results are not statistically significant. The ratio of Neuropil to Kenyon cell (N:K) was also higher in mature males than in newly eclosed males (Fig. 2a; t =  − 4.32, df = 12, p = 0.001, Hedges’ *g* = 2.34). In *B. impatiens*, mature males had 24.5% larger calyces (Fig. 1b; t =  − 4.34, df = 16, p = 0.0005, Hedges’ *g* = 2.08), and 19.5% larger mushroom body neuropil (Fig. 1b; t =  − 3.84, df = 16, p = 0.001, Hedges’ *g* = 1.85) than newly eclosed males. *Bombus impatiens* N:K ratios were significantly higher in mature males than in newly eclosed males (Fig. 2b; t =  − 5.12, df = 16, p = 0.0001, Hedges’ *g* = 2.48). Kenyon cells were not significantly different in relative volume between mature and newly eclosed males of either species (Fig. 1a,b; *M. genalis:* t = 1.20, df = 12, p = 0.255, Hedges’ *g* =  − 0.65; *B. impatiens*: t = 1.25, df = 16, p = 0.228, Hedges’ *g* =  − 0.75). Likewise, mushroom body lobes relative volume were not significantly different between mature and newly eclosed males of either species (Fig. 1a,b; *M. genalis:* t =  − 1.41, df = 12, p = 0.185, Hedges’ *g* = 0.76 and *B. impatiens*: t =  − 1.42, df = 16, p = 0.175, Hedges’ *g* = 0.69). Results for calyx volume were similar when normalized to Kenyon cell volume instead of whole brain, such that the calyces were larger in mature males in both species, and mushroom body lobes were significantly enlarged in mature *B. impatiens* males when normalized to Kenyon cell volume (see Supplementary Fig. S2 online).

## Discussion

We found strikingly similar patterns of age-related neuroplasticity in *M. genalis* and *B. impatiens* males. In both species, expansion of mushroom body structures, including calyx and neuropil, occurred with age under experimental conditions void of ecologically-relevant experience. Our work adds to the sparse literature on volumetric neuroplasticity in Hymenopteran males while improving our general understanding of potential functions of experience-expectant neuroplasticity in insects.

Our results provide the first definitive evidence that large-scale volumetric neuroplasticity is driven by age, independent of relevant experience (e.g., social, flight, etc.), in male Hymenoptera. Previous research has documented mushroom body expansion associated with the combination of maturation and experience in paper wasp^14^ and honey bee^13^ males. Similar to our results, paper wasp and honey bee calyx and neuropil volumes increase, respectively, as males mature. Developmental maturation was observed with flight initiation, social interaction, and mating behavior in these species^13,14^. But, since the individual effects of age and experience were not experimentally controlled for in these previous studies, they could not be evaluated independently. Mature males in our study were experimentally deprived of flight, social cues, and mating experience. We aimed to isolate experience-independent from experience-dependent brain changes during adult development. However, social deprivation can adversely affect eusocial insects, leading to impaired brain development, learning, and behaviors^30–32^. Therefore, we cannot eliminate the possibility that the volumetric plasticity observed may include effects associated with unnatural rearing conditions, inadvertent stress, or unidentified experiences. Nevertheless, our results show that, across multiple species with different rearing conditions, male brains change relatively consistently with age, independent of ecologically-relevant experience, suggesting that age-related neuroplasticity may be common in male Hymenoptera.

Age-related mushroom body expansion coincides with reproductive maturation in male bees, and may represent a common developmental change associated with dispersal from the nest prior to the onset of mating. A primary function of male Hymenoptera is to inseminate a female(s)^17,33–37^, and experience-expectant neuroplasticity may potentially facilitate this behavior. Our approach cannot identify precisely when age-related brain changes occurred in either species. However, bumble bee (*B. vosnesenskii*) males reach reproductive maturity by 8–10 days post-eclosion^38^, and we observed significant mushroom body expansion in *B. impatiens* males after 10 d of aging. Scent-marking and patrolling is the most common pre-mating strategy in *Bombus*^27^, whereby males pheromonally mark points along a flight route^27,39,40^. Patrolling is similar to “trap-line” foraging^27^, which is associated with the phylogenetic expansion of mushroom bodies in *Heliconius* butterfly species that also exhibit age-related brain plasticity^41^. Female bumble bees utilize learned aspects of their environment for spatial orientation^23,24^, and males have learning capabilities equivalent to females^42^; therefore, while speculative, the brain changes observed with *Bombus* male maturation may be an important preparation for the potential cognitive challenges related to mate finding behaviors. Similarly, we found that mature (6-d old) sweat bee males had enlarged mushroom bodies relative to newly eclosed males. The mating behavior of *M. genalis* is unknown. However, males typically stay in their natal nest for up to 4 d past emergence^28^, during which time they are fed via trophallaxis by their mothers and sisters^43^. It is presumably during this time that they are becoming reproductively mature. The males of some Halictine species exhibit mate patrolling^44^, but it is unknown whether *M. genalis* conduct these behaviors. In honey bees, neuropil expansion coincides with the time that males reach sexual maturity (6–12 d)^13,45,46^. Age-related neuroplasticity observed in male paper wasps (*M. mastigophorus*) may also coincide with reproductive maturity^14^. Males of this species are atypical of other social insects in that they remain on their natal nests long after eclosion, departing only temporarily to mate^47,48^. The age at first nest departure (median = 5 d^49^), however, is still comparable with those of the bees studied here. Our study was not designed to identify the functional relevance of age-related brain development in males, but instead provides new insight for subsequent work. Thus, while the functional roles of age-related plasticity remain unclear, our study and previous studies suggest that the age-related neuroplasticity observed within males across species may be associated with departing the nest in search of mating opportunities—a predictably timed, common event driving the male life-cycle. This pattern of expansion is similar to the ‘experience-expectant’ neuroplasticity observed in the females of some, but not all, social insects.

Our results also suggest that intraspecific sex differences in age-related neuroplasticity patterns can occur among some social insect species. Neuroanatomical changes in the female workers of highly social bees accompany shifts in colony needs^6–11^, coinciding with age-related behavioral transitions from working inside the hive to foraging^6,9,50^. Yet, this age-related task specialization is not universal across social species. In bumble bees, where division of labor is size-based instead of age-related, females exhibit mushroom body expansion within the first few days of life^12,51,52^, which accompanies their capacity for behavioral maturation soon after emergence^53^. These changes are similar to those observed in our mature *B. impatiens* males. However, experience-expectant neuroplasticity is absent in *M. genalis* females^54^ (though see^55^), which also lack age-related task specialization^25,56^. Our finding that mushroom body expansion occurs with maturation in male *M. genalis* suggests that experience-expectant neuroplasticity can occur in males, even when it is absent in females. Future work comparing sex-specific patterns of brain development in additional bee species is needed to determine the pervasiveness of intersexual differences in neuroplasticity. Investigating socioecological drivers of neuroplasticity in both sexes, particularly in solitary species where females lack age-related plasticity^57,58^, will provide a more robust understanding of the relationship between neuroplasticity and social evolution.

## Methods

### Field collections and laboratory rearing

We conducted the experiment for *Megalopta genalis* from March to May 2015 on Barro Colorado Island (BCI), Republic of Panama. Twice daily—once in the morning and evening—we collected newly eclosed males from their natal nests, which consisted of dead sticks or branches^25^. We randomly assigned newly eclosed males to either ‘newly eclosed’ or ‘mature’ treatment groups. Bees designated as ‘newly eclosed’ (*N* = 8) were sacrificed within minutes, whereas ‘mature’ (*N* = 6) males were housed individually in food storage containers for 6 d in an incubator (27 °C, 70%, 0:24 L:D) and provided food (36% sugar, 7% protein w/v) ad libitum. Food was mixed by dissolving six Nature’s Blend Protein tablets (National Vitamin Company, Casa Grande, AZ) in 50 ml distilled water and changed twice daily.

During August to December 2018, we produced *B. impatiens* males from queenless microcolonies (*N* = 11) generated using three commercial colonies from Koppert Biological Systems (Howell, MI, USA). Microcolonies consisted of five *B. impatiens* workers from the same source colony that were housed in custom rearing cages: 173 × 130 × 91 mm food storage containers that included aluminum mesh bottoms and hinged plexiglass tops. We supplied microcolonies with 50% sugar water (cane sugar dissolved in distilled water) supplemented with potassium sorbate, citric acid, Honey B Healthy Essential Oil, and Honey B Healthy Amino Boost, as well as pollen dough ad libitum. Our pollen dough consisted of honey bee collected pollen (Betterbee, Greenwich, NY, USA) mixed with the aforementioned sugar water until it reached a consistency similar to moist, slightly tacky fine-grained sand. We stored microcolonies in an incubator maintained at 27 °C and ~ 60–70% relative humidity on a 16:8 h light:dark cycle. Male brood require ~ 24 d to develop before eclosion^59^. We checked brood development and for newly eclosed males daily. After eclosion, we randomly assigned new males to one of two treatment groups: ‘newly eclosed’ (*N* = 11) males were sacrificed immediately, whereas ‘mature’ (*N* = 7) males were maintained individually for 10 d in the rearing cages and conditions described above.

Where applicable, we followed the recommended guidelines for animal care and use^60^.

### Preservation and dissection

For each species, we terminated males via decapitation after immobilizing them on ice for ~ 5 min. We removed eye capsules and mouthparts to facilitate preservation. Head capsules were preserved in 4% zinc paraformaldehyde (PFA) and 4% PFA for *M. genalis* and *B. impatiens*, respectively, and then stored at 4 °C until dissection. Prior to dissection, we rinsed head capsules in 1X phosphate-buffered saline (PBS; 3 × 10 min), followed by brain dissections in 1 × PBS. Dissected brains were post-fixed in 2% glutaraldehyde at room temperature for 2 d. After 48 h, brains were rinsed (1X PBS; 3 × 10 min), formamide bleached for ~ 30–45 min to remove residual pigment (1 × PBS, 3% formamide, 1% triton-X, and 20% hydrogen peroxide) (modified protocol from^61^), rinsed again (1X PBS; 3 × 10 min), and then serially dehydrated via a series of ascending ethanol concentrations (30%, 50%, 70%, 90%, 95%, and 3 × 100%, 10 min each). Lastly, we cleared and stored brains in methyl salicylate at − 20 °C until imaging.

### Confocal microscopy and structure tracing

We imaged brains using autofluorescence on a laser confocal microscope (Zeiss LMS 710). Whole brains were mounted in methyl salicylate and scanned as z-stack series ranging from 760 to 925 µm thick. We imaged brains as 3 × 2 tile scans (2867 × 1946 pixels) with optical slices captured in 5 µm intervals. For both species, brains were imaged simultaneously using two lasers, though the wavelengths, laser power, and gains varied by species. We imaged *M. genalis* at 410–484 nm and 495–538 nm wavelengths, 3.5 and 3.0 power, and 504–535 and 495–517 gains for laser 1 and 2, respectively. For *B. impatiens*, the first laser had a wavelength between 410 and 485 nm, a laser power of 4.0, and a gain range between 527 and 567. The second laser had a wavelength, power, and gain range of 495–538 nm, 3.5, and 518–558. Whole brain image stacks were saved as individual jpegs.

**Figure 3:**
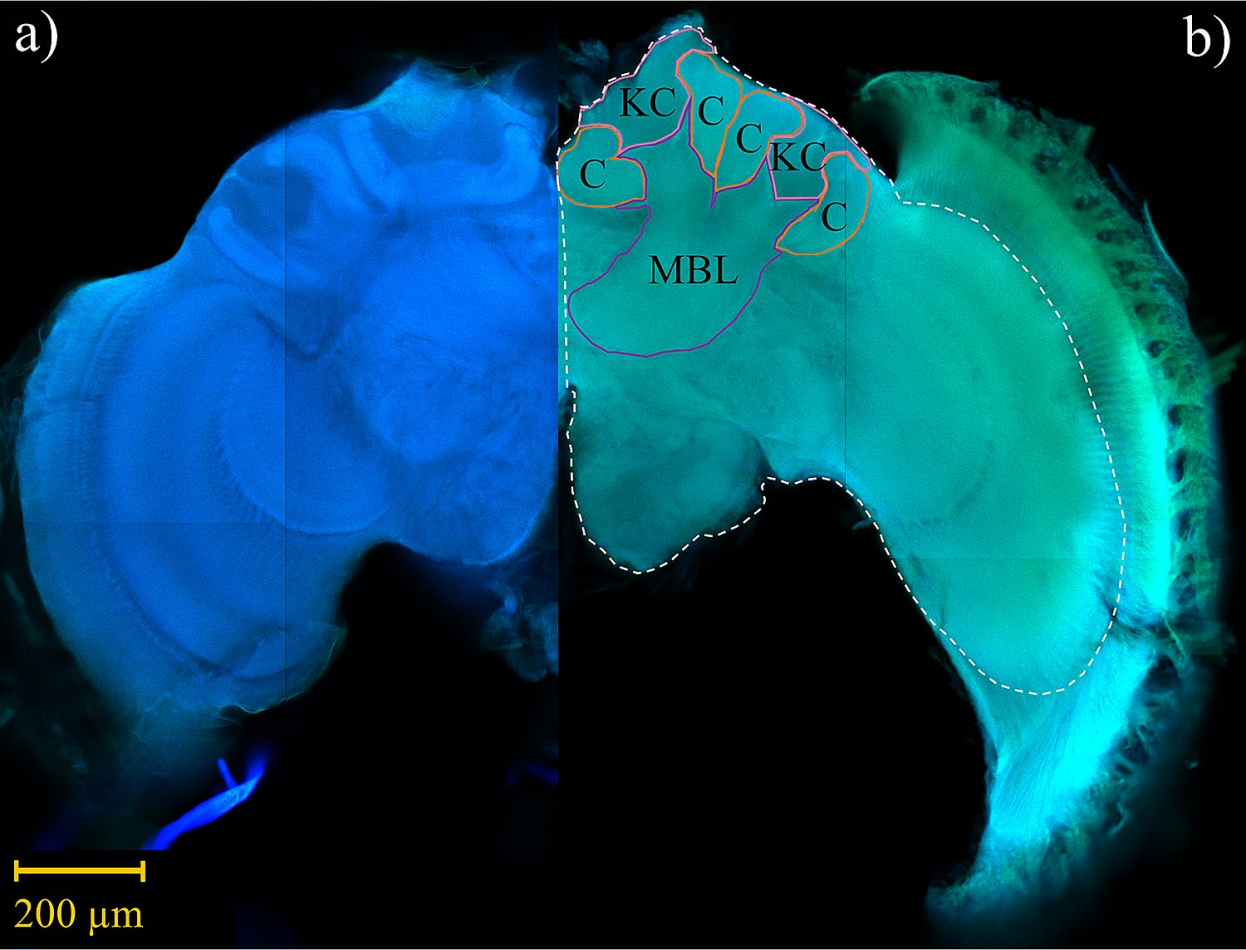
Confocal microscope image of a (**a**) *Bombus impatiens* and (**b**) *Megalopta genalis* male brain. Image captures are individual slices taken from raw image stacks. Volumetric measurements were assessed for mushroom body calyces (C; lip, collar, and basal ring as one structure), Kenyon cells (KC), and mushroom body lobes (MBL; peduncle and lobes as one structure). Solid contour lines include structure-specific boundaries (pink (KC), orange (C), and purple (MBL)), whereas the dotted white line indicates the boundary for a whole brain trace. Scale bar 200 µm.

Throughout confocal stacks, we traced individual structures on every other optical slice (10 µm intervals) and estimated volumetric measurements via serial reconstruction using Reconstruct software (Fiala^62^; version 1.1.0.0; available at http://synapses.clm.utexas.edu). Due to occasional tissue damage, we traced each structure unilaterally to maximize sample inclusion. For undamaged brains, we randomly selected either the right or left side to trace, whereas undamaged sides were always traced for brains with tissue damage. The number of right and left side brain traces were distributed similarly across treatment group (*M. genalis*: Yates corrected *X*^2^ (1, N = 14) = 0.29, p = 0.589 and *B. impatiens*: Yates corrected *X*^2^ (1, N = 16) = 0.02, p = 0.896). Whole brain traces were also conducted unilaterally, corresponding with the side used for structure tracing, and always excluded the lamina and retina (Fig. 3). We conducted all confocal imaging, tracing, and 3D reconstruction without knowledge of the experimental treatment group to which each sample belonged.

The structures examined included the calyces (lip, collar, and basal ring as one structure), mushroom body lobes (peduncle and lobes as one structure), total neuropil (calyces and mushroom body lobes), and Kenyon cells (Fig. 3). Neuropil to Kenyon cell volumetric increases can occur with age-related plasticity^6,7,29,57^; therefore, we also assessed neuropil:Kenyon cell ratios (N:K). For each sample, we standardized volumes using two methods (see Supplementary Information online): 1) structure volumes to whole brain, referred to as “relative volumes”, and 2) structure volumes to Kenyon cells (results reported in Supplementary Information online).

### Statistical analyses

All statistical analyses were conducted in *R* version 4.0.4 (https://www.r-project.org/). We assessed the relative volumes (structure:wholebrain), structure:KC , and N:K ratios using Student’s t-tests (*stats*, version 4.0.4) to compare ‘newly eclosed’ and ‘mature’ bees independently for each species. We used visual inspection of qq-plots (*car*, version 3.0–10; Fox and Weisberg^63^) and Anderson–Darling normality tests (*Nortest*, version 1.0–4; https://CRAN.R-project.org/package=nortest) to verify normality assumptions. One variable—relative calyx volume for *B. impatiens*—violated normality assumptions, so we applied a Box-Cox transformation (*MASS*, version 7.3–53; Venables and Ripley^64^) using λ =  − 1.455. We assessed homogeneity of variance using *R* package *car* (version 3.0–10; Fox and Weisberg^63^). Relative Kenyon cell volume violated variance assumptions, therefore we conducted a second Box-Cox transformation using λ =  − 0.970. We determine effect size between groups by calculating Hedges’ *g* (*effsize*, version 0.8.1, Torchiano^65^). To account for multiple comparisons, we applied a Bonferroni correction and adjusted statistical significance to α = 0.01.

### Data and code availability

The data are available on Dryad (Hagadorn et al.^66^) and code is stored in GitHub: https://github.com/kapheimlab/male_neuroplasticity.

## Supplementary information

Supplementary Information.

## References

[1] Kolb B, Gibb R, Stuss D, Winocur G, Robertson I (2008). Cognitive Neurorehabilitation: Evidence and Application.

[2] Frankenhuis WE, Nettle D (2020). Integration of plasticity research across disciplines. Curr. Opin. Behav. Sci..

[3] Greenough WT, Black JE, Wallace CS (1987). Experience and brain development. Child Dev..

[4] Gronenberg W, Heeren S, Hölldobler B (1996). Age-dependent and task-related morphological changes in the brain and the mushroom bodies of the ant *Camponotus floridanus*. J. Exp. Biol..

[5] Seid M, Harris K, Traniello J (2005). Age-related changes in the number and structure of synapses in the lip region of the mushroom bodies in the ant *Pheidole dentata*. J. Comp. Neurol..

[6] Withers GS, Fahrbach SE, Robinson GE (1993). Selective neuroanatomical plasticity and division of labour in the honeybee. Nature.

[7] Withers GS, Fahrbach SE, Robinson GE (1995). Effects of experience and juvenile hormone on the organization of the mushroom bodies of honey bees. J. Neurobiol..

[8] Fahrbach SE, Moore D, Capaldi EA, Farris SM, Robinson GE (1998). Experience-expectant plasticity in the mushroom bodies of the honeybee. Learn. Mem..

[9] Farris SM, Robinson GE, Fahrbach SE (2001). Experience- and age-related outgrowth of intrinsic neurons in the mushroom bodies of the adult worker honeybee. J. Neurosci..

[10] Durst C, Eichmüller S, Menzel R (1994). Development and experience lead to increased volume of subcompartments of the honeybee mushroom body. Behav. Neural Biol..

[11] Tomé HVV, Rosi-Denadai CA, Pimenta JFN, Guedes RNC, Martins GF (2014). Age-mediated and environmentally mediated brain and behavior plasticity in the stingless bee Melipona quadrifasciata anthidioides. Apidologie.

[12] Jones B, Leonard A, Papaj D, Gronenberg W (2013). Plasticity of the worker bumblebee brain in relation to age and rearing environment. Brain Behav. Evol..

[13] Fahrbach SE, Giray T, Farris SM, Robinson GE (1997). Expansion of the neuropil of the mushroom bodies in male honey bees is coincident with initiation of flight. Neurosci. Lett..

[14] Molina Y, O’Donnell S (2008). Age, sex, and dominance-related mushroom body plasticity in the paperwasp *Mischocyttarus mastigophorus*. Dev. Neurobiol..

[15] O’Donnell S, Donlan NA, Jones TA (2004). Mushroom body structural change is associated with division of labor in eusocial wasp workers (Polybia aequatorialis, Hymenoptera: Vespidae). Neurosci. Lett..

[16] Gronenberg W, Riveros AJ, Gadau J, Fewell J (2009). Organization of Insect Societies: From Genome to Sociocomplexity.

[17] Beani L, Dessì-Fulgheri F, Cappa F, Toth A (2014). The trap of sex in social insects: from the female to the male perspective. Neurosci. Biobehav. Rev..

[18] Fletcher DJ, Michener C (1987). Kin recognition in animals.

[19] Wcislo WT (1987). The role of learning in the mating biology of a sweat bee Lasioglossum zephyrum (Hymenoptera: Halictidae). Behav. Ecol. Sociobiol..

[20] Barrows EM, Bell WJ, Michener CD (1975). Individual odor differences and their social functions in insects. Proc. Natl. Acad. Sci. USA.

[21] Barrett M (2021). Neuroanatomical differentiation associated with alternative reproductive tactics in male arid land bees, *Centris pallida* and *Amegilla dawsoni*. J. Comp. Physiol. A.

[22] Woodgate JL (2021). Harmonic radar tracking reveals that honeybee drones navigate between multiple aerial leks. iScience.

[23] Sovrano VA, Potrich D, Vallortigara G (2013). Learning of geometry and features in bumblebees (Bombus terrestris). J. Comp. Psychol..

[24] Sovrano VA, Rigosi E, Vallortigara G (2012). Spatial reorientation by geometry in bumblebees. PLoS ONE.

[25] Wcislo WT (2004). The evolution of nocturnal behaviour in sweat bees, *Megalopta genalis* and *M. ecuadoria* (Hymenoptera: Halictidae): An escape from competitors and enemies?. Biol. J. Linn. Soc..

[26] Smith AR, Kapheim KM, Kingwell CJ, Wcislo WT (2019). A split sex ratio in solitary and social nests of a facultatively social bee. Biol. Lett..

[27] Goulson D (2010). Bumblebees: Behaviour, Ecology, and Conservation.

[28] Kapheim KM, Smith AR, Nonacs P, Wcislo WT, Wayne RK (2013). Foundress polyphenism and the origins of eusociality in a facultatively eusocial sweat bee, Megalopta genalis (Halictidae). Behav. Ecol. Sociobiol..

[29] Fahrbach SE (2006). Structure of the mushroom bodies of the insect brain. Annu. Rev. Entomol..

[30] Seid MA, Junge E (2016). Social isolation and brain development in the ant *Camponotus floridanus*. Sci. Nat..

[31] Cabirol A, Brooks R, Groh C, Barron AB, Devaud J-M (2017). Experience during early adulthood shapes the learning capacities and the number of synaptic boutons in the mushroom bodies of honey bees (*Apis mellifera*). Learn. Mem..

[32] Maleszka J, Barron AB, Helliwell PG, Maleszka R (2009). Effect of age, behaviour and social environment on honey bee brain plasticity. J. Comp. Physiol. A.

[33] Boomsma JJ, Baer B, Heinze J (2005). The evolution of male traits in social insects. Annu. Rev. Entomol..

[34] Heinze J, Schrempf A (2008). Aging and reproduction in social insects—A mini-review. Gerontology.

[35] Hrassnigg N, Crailsheim K (2005). Differences in drone and worker physiology in honeybees (Apis mellifera). Apidologie.

[36] Wilson EO (1971). The Insect Societies.

[37] Michener CD (1974). The Social Behavior of the Bees.

[38] Herndon, J. D. Investigating nest box utilization by bumble bees and reproductive development of male bumble bees. Master's thesis. 10.26076/2256-306b (2020).

[39] Alford DV (1975). Bumblebees.

[40] Valterová I, Martinet B, Michez D, Rasmont P, Brasero N (2019). Sexual attraction: A review of bumblebee male pheromones. Z. Naturforschung C.

[41] Montgomery SH, Merrill RM, Ott SR (2016). Brain composition in Heliconius butterflies, posteclosion growth and experience-dependent neuropil plasticity. J. Comp. Neurol..

[42] Muth F, Tripodi AD, Bonilla R, Strange JP, Leonard AS (2021). No sex differences in learning in wild bumblebees. Behav. Ecol..

[43] Kapheim KM, Chan T-Y, Smith A, Wcislo WT, Nonacs P (2016). Ontogeny of division of labor in a facultatively eusocial sweat bee *Megalopta genalis*. Insectes Soc..

[44] Barrows EM (1976). Mating behavior in halictine bees (Hymenoptera: Halictidae): I, patrolling and age-specific behavior in males. J. Kans. Entomol. Soc..

[45] Snodgrass RE (1956). Anatomy of the Honey Bee.

[46] Harbo JR, Rinderer TE (1986). Bee Genetics and Bee Breeding.

[47] O’Donnell S (1999). The function of male dominance in the Eusocial wasp, *Mischocyttarus mastigophorus* (Hymenoptera: Vespidae). Ethology.

[48] O’Donnell S, Fiocca K, Congdon R (2021). Social network analysis of male dominance in the paper wasp *Mischocyttarus mastigophorus* (Hymenoptera: Vespidae). J. Insect Behav..

[49] Molina Y, O’Donnell S (2009). Males exhibit novel relationships of dominance with nest departure in the social paper wasp *Mischocyttarus mastigophorus* (Hymenoptera: Vespidae). Ethology.

[50] Ismail N, Robinson GE, Fahrbach SE (2006). Stimulation of muscarinic receptors mimics experience-dependent plasticity in the honey bee brain. Proc. Natl. Acad. Sci. USA.

[51] Kraft N, Spaethe J, Rössler W, Groh C (2019). Neuronal plasticity in the mushroom-body calyx of bumble bee workers during early adult development. Dev. Neurobiol..

[52] Riveros AJ, Gronenberg W (2010). Brain allometry and neural plasticity in the bumblebee *Bombus occidentalis*. Brain Behav. Evol..

[53] Heinrich B (2004). Bumblebee Economics.

[54] Jaumann S, Seid MA, Simons M, Smith AR (2019). Queen dominance may reduce worker mushroom body size in a social bee. Dev. Neurobiol..

[55] Smith AR, Seid MA, Jimenez LC, Wcislo WT (2010). Socially induced brain development in a facultatively eusocial sweat bee *Megalopta genalis* (Halictidae). Proc. R. Soc. B Biol. Sci..

[56] Smith AR, Wcislo WT, O’Donnell S (2007). Survival and productivity benefits to social nesting in the sweat bee *Megalopta genalis* (Hymenoptera: Halictidae). Behav. Ecol. Sociobiol..

[57] Withers GS, Day NF, Talbot EF, Dobson HE, Wallace CS (2008). Experience-dependent plasticity in the mushroom bodies of the solitary bee *Osmia lignaria* (Megachilidae). Dev. Neurobiol..

[58] Hagadorn MA, Johnson MM, Smith AR, Seid MA, Kapheim KM (2021). Experience, but not age, is associated with volumetric mushroom body expansion in solitary alkali bees. J. Exp. Biol..

[59] Cnaani J, Schmid-Hempel R, Schmidt JO (2002). Colony development, larval development and worker reproduction in *Bombus impatiens* Cresson. Insectes Soc..

[60] Percie du Sert N (2020). The ARRIVE guidelines 2.0: Updated guidelines for reporting animal research. J Cerebr. Blood F. Met..

[61] Zukor KA, Kent DT, Odelberg SJ (2010). Fluorescent whole-mount method for visualizing three-dimensional relationships in intact and regenerating adult newt spinal cords. Dev. Dyn..

[62] Fiala JC (2005). Reconstruct: A free editor for serial section microscopy. J. Microsc..

[63] Fox J, Weisberg S (2019). An R Companion to Applied Regression.

[64] Venables WN, Ripley BD (2002). Modern Applied Statistics with S.

[65] Torchiano, M. Effsize—A package for efficient effect size computation v. 0.8.1. 10.5281/zenodo.196082 (2016).

[66] Hagadorn MA (2021). Data from: Age-related mushroom body expansion in male sweat bees and bumble bees data sets. Dryad.

