# Supplementary material for: Age-related mushroom body expansion in male sweat bees and bumble bees
Source: Sci Rep. 2021 Aug 23;11:17039. doi: 10.1038/s41598-021-96268-w (PMC8382693; doi:10.1038/s41598-021-96268-w)
Supplement: Supplementary file 1 — Supplementary Information. [file 41598_2021_96268_MOESM1_ESM.pdf]

Supplementary Materials for:

Age-related mushroom body expansion in male sweat bees and bumble bees

Mallory A. Hagadorn[1*], Karlee Eck[1], Matthew Del Grosso[1†], Xavier Haemmerle[1], William T. Wcislo[2], and Karen M. Kapheim[1, 2*]

[1]Department of Biology, Utah State University, 5305 Old Main Hill, Logan, UT 84322, USA.

[2]Smithsonian Tropical Research Institute, 0843-03092 Panama City, Republic of Panama.

[†]Deceased: 21 May 2015

[*]Corresponding Authors: Mallory A. Hagadorn; USA; Email: mallory.hagadorn@usu.edu and Karen M. Kapheim; USA; Email: karen.kapheim@usu.edu

Alternative Scaling Methods

Whole brain volumes for *M. genalis*, were not significantly different between groups (Supplementary Fig.S1; $t = 1.14$, $df = 12$, $p = 0.28$). However, mature *B. impatiens* males had significantly larger (23.8%) whole brain volumes compared to newly-eclosed individuals (Supplementary Fig. S1; $t = -3.08$, $df = 16$, $p = 0.007$). We investigated potential methodical factors than may have affected whole brain volumes using Spearman's rho correlation analysis. These results suggested no relationship among whole brain volume and the duration (in days) of storage in PFA before dissection ($r_s = -0.34$, $p = 0.172$), number of days between dissection and imaging ($r_s = 0.08$, $p = 0.753$), and when brains were traced ($r_s =-0.11$, $p = 0. 654$). Thus, in both species, we also normalized the absolute calyx and mushroom body lobe volumes to the Kenyon cells (calyx:KCs and mblobes:KCs; sensu [1,2]) to verify results. We applied a Bonferroni correction for multiple comparisons and adjusted the significance threshold to $\alpha = 0.025$. Calyx:Kenyon cells volume of mature males was significantly larger than newly-eclosed males in both *M. genalis* (Supplementary Fig. S2; $t = -4.83$, $df = 12$, $p = 0.0004$, Hedges' $g = 2.61$) and *B. impatiens* (Supplementary Fig. S2; $t = -4.91$, $df = 16$, $p = 0.0002$, Hedges' $g = 2.37$). Mature *B. impatiens* males also had enlarged (26.6%) MB lobe:Kenyon cell volume relative to newly-eclosed bees (Supplementary Fig. S2; $t = -3.11$, $df = 16$, $p = 0.01$, Hedges' $g = 1.50$). A 20.5% increase in MB lobe:Kenyon cell volume was also observed in mature *M. genalis* males, but the difference is not significant (Supplementary Fig. S2; $t = -2.13$, $df = 12$, $p = 0.055$, Hedges' $g = 1.15$).

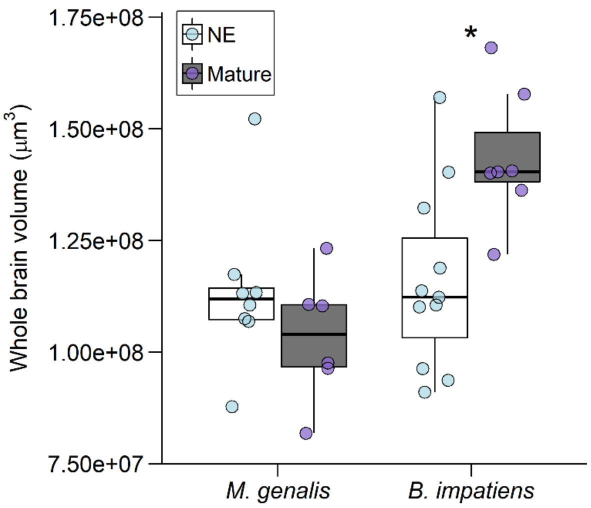

Supplementary Figure S1. Whole brain volumes by species. Dots represent individual data points for newly-eclosed (NE; white boxes; light blue dots) and mature (gray boxes; light purple dots) males. "*" = unadjusted $p < 0.05$. Boxes represent the interquartile range, with the lines as medians. Whiskers extend to 1.5 the interquartile range.

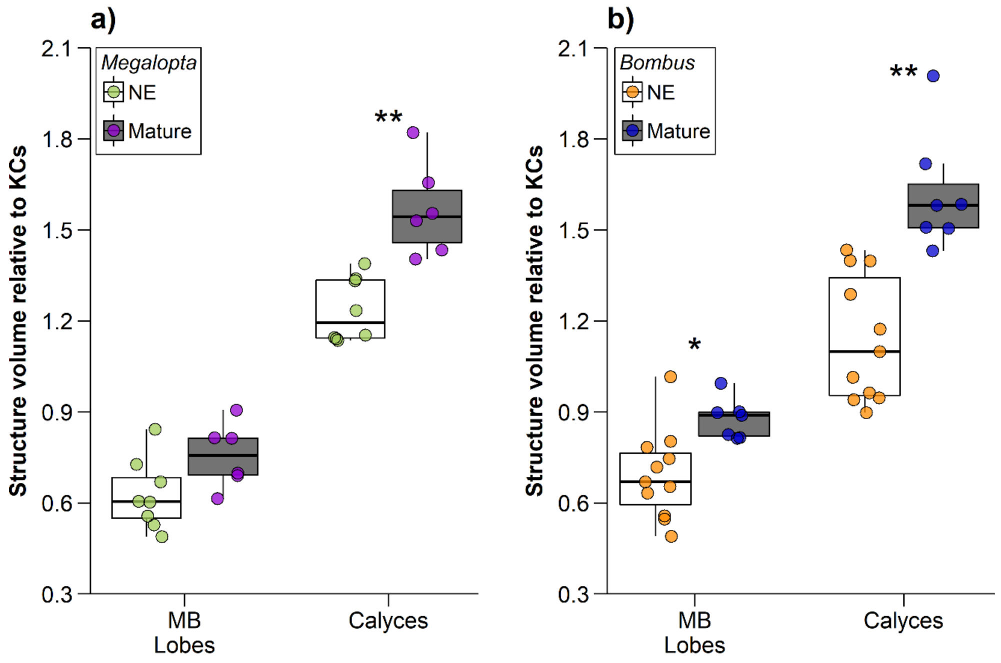

Supplementary Figure S2. Age-related neuroplasticity for mushroom body structures standardized to Kenyon cell (KC) volumes. Mushroom body lobes and calyx proportions for a) *Megalopta genalis* and b) *Bombus impatiens*. Dots represent individual data points for newly-eclosed (NE; white boxes; *M. genalis* = green dots; *B. impatiens* = yellow dots) and mature (gray boxes; *M. genalis* = purple dots; *B. impatiens* = blue dots) males. "*" = unadjusted $p < 0.05$ and "**" = unadjusted $p < 0.001$. Boxes indicate interquartile range, lines are medians, and whiskers extend to 1.5 the interquartile range.

References

1. Molina, Y. & O'Donnell, S. Age, sex, and dominance-related mushroom body plasticity in the paperwasp *Mischocyttarus mastigophorus*. *Dev. Neurobiol.* **68**, 950-959, https://doi.org/10.1002/dneu.20633 (2008).

2. Molina, Y. & O'Donnell, S. Mushroom body volume is related to social aggression and ovary development in the paperwasp *Polistes instabilis*. *Brain Behav. Evol.* **70**, 137-144 (2007).
